# Simulation on two-phase refrigerant compression in the cylinder of rotary compressors using CFD method

**DOI:** 10.1038/s41598-024-56856-y

**Published:** 2024-03-13

**Authors:** Nini Guo, Jie Lin, Jianhua Wu

**Affiliations:** 1 College of Mechanical Engineering, Quzhou University, Quzhou, 324000 China; 2 School of Energy and Power Engineering, Xi’an Jiaotong University, Xi’an, 710049 China

**Keywords:** Rotary compressor, Two-phase compression, CFD, R290, Mechanical engineering, Fluid dynamics

## Abstract

The two-phase compression process in the rotary compressor often occurs, such as defrosting and startup processes, which has a significant impact on the performance and reliability of air conditioning systems. In this paper, the CFD simulations predicting the two-phase refrigerant compression process in the compressor cylinder are conducted using the commercial software ANSYS Fluent. The dynamic mesh for the fluid domain and phase change model for the refrigerant are considered in the simulation. Effects of initial liquid volume fraction, refrigerant type and compressor type on the two-phase compression characteristics using R290 as refrigerant are carried out. Variations of the pressure, temperature, gas fraction distribution and evaporation rate in the cylinder are discussed. The results show that most liquid accumulates near the leakage gap and the bottom of the compression chamber during the two-phase compression process. The peak pressure during the two-phase compression decreases with the increase of the liquid volume fraction. The evaporation rate of R32 in the cylinder is much higher than that of R290. The maximum pressure of the reciprocating compressor is 2.26 times higher than that of the rotary compressor.

## Introduction

The rotary compressor is one of the key components of the residential air conditionings, and it is the main factor affecting the performance and reliability of the air-conditioning system. Moreover, it is also used more and more widely in other household applications, such as dehumidifiers, heat pump water heaters, clothes dryers, etc. However, under some operating conditions, such as low temperature start-up, defrosting, liquid injection to improve the performance and decrease the discharge temperature, liquid refrigerant or oil inevitably enter the compression chamber of the compressor^1–4^, resulting in the wet compression in the cylinder. During the wet compression process, also named liquid slugging, the pressure in the cylinder surges to much higher than that of normal value, causing irreversible damage to the crankshaft and other components of the compressor. Especially for new refrigerants, such as R290, the research on wet compression characteristics of R290 in the rotary compressor is necessary because of its special thermophysical properties.

Because the excessive pressure in the cylinder during liquid compression threatens the compressor reliability, many studies have been conducted to predict and reveal the law of liquid compression using experimental and empirical formula method. Singh^5^ experimentally studied the pressure overload in the cylinder caused by liquid compression using refrigerant R22. Peak pressure up to 8.37 MPa was measured during the startup of the compressor. A model considering the compression process with a separated gas and liquid media in the cylinder was proposed to predict the phenomenon. Simpson^6^ devised several different methods of measuring cylinder pressures generated during liquid slugging in compressors. He also presented an empirical relation for predicting maximum pressure as a function of compressor structures. Through the analysis of the experiment results, Laughman^7^ found that the overpressure generated by liquid compression could be effectively predicted by observing the fluctuation of power consumption. Prasad^8^ used a polytropic index method to predict the instantaneous liquid slugging pressure in the cylinder of a reciprocating compressor.

Because all the factors are included in the polytropic index for the empirical formula method, the thermodynamic process mechanism during the two-phase compression in the cylinder can't be accurately revealed. The homogeneous model based on energy and mass conservation equations was proposed by many researchers. Liu^9^ derived the thermodynamic equation to simulate the two-phase refrigerant compression process in a rotary compressor. This paper also discussed the influence of compressor type on liquid slugging. Dutta^10^ studied the variation of temperature and pressure in the cylinder of a rotary compressor during two-phase compression process by using three models, homogeneous model, liquid slugging model and droplet model. The accuracy of homogeneous flow model was experimentally verified. Because the homogeneous flow model can’t well predict the peak pressure in cylinder during liquid compression process. Lin^11^ proposed a novel two-phase mathematic model, which considers refrigerant phase change, kinetic energy and heat exchange between refrigerant and the wall, to predict the pressure jump in the cylinder under liquid compression conditions. Effects of rotation speed initial, gas fraction and refrigerant type on the liquid compression were investigated. Although these mathematical models can effectively predict the overall thermal process changes in the compression chamber, they are all zero-dimensional models based on the homogeneous flow assumptions. The momentum equation term is not contained in the equations. Therefore, the liquid slugging, especially the local state changes or distribution of the refrigerant in the cylinder, cannot be accurately predicted.

With the development and application of CFD (Computational Fluid Dynamics) software, some scholars have conducted numerical simulation analysis of two-phase compression in compressors by using commercial software. Riccardo^12^ used the software of ANSYS fluid–structure coupling method to simulate gas–liquid two-phase compression and discharge characteristics of hydrocarbon refrigerants in the reciprocating compressor. The results showed that liquid presence in the cylinder may cause up to five times the normal pressure on the compressor. Ma^13^ simulated the transient liquid distribution and pressure fluctuation in the cylinder of reciprocating multiphase pump with different gas models and gas fractions. Through the calculations and experimental analysis, the impacts of liquid slugging on the reciprocating compressor can be predicted. Wang^14^ used the commercial CFD software package Fluent to numerically simulated the working process for a multiphase scroll pump. The grid generation and numerical methods were discussed. The simulation results suggested the key parameter values for design of multiphase scroll pumps. Hsu^15^ used the CFD technique to simulate flow structure of the rotary compressor. The pressure, temperature, and mass flow rate of refrigerant were obtained for analyses the efficiency of compressor.

The two-phase compression characteristics in the compression chamber of rotary compressors have been fully studied using the methods of experimental measurements, empirical formulas and thermodynamic methods. However, these methods cannot reveal the variations of the pressure, temperature and liquid distribution in the cylinder of rotary compressors, provide guidance on the design and control of the two-phase compression.

In this paper, the two-phase compression characteristics in the compression chamber of the rotary compressors are numerically simulated using the CFD method. The numerical simulations are conducted using the commercial CFD software package Fluent by choosing the Euler two-phase flow calculation model. Effects of initial liquid volume fraction, refrigerant type and compressor type on two-phase compression in the cylinder are discussed using R290 as refrigerant. The variations of temperature, pressure, evaporation rate and liquid distribution with crank angle are analyzed. The research in this paper will help to reduce the risk of liquid slugging and improve two-phase compression performance of the rotary compressor in the air conditioning systems.

## Modeling

### Working principle and geometry model

**Figure 1:**
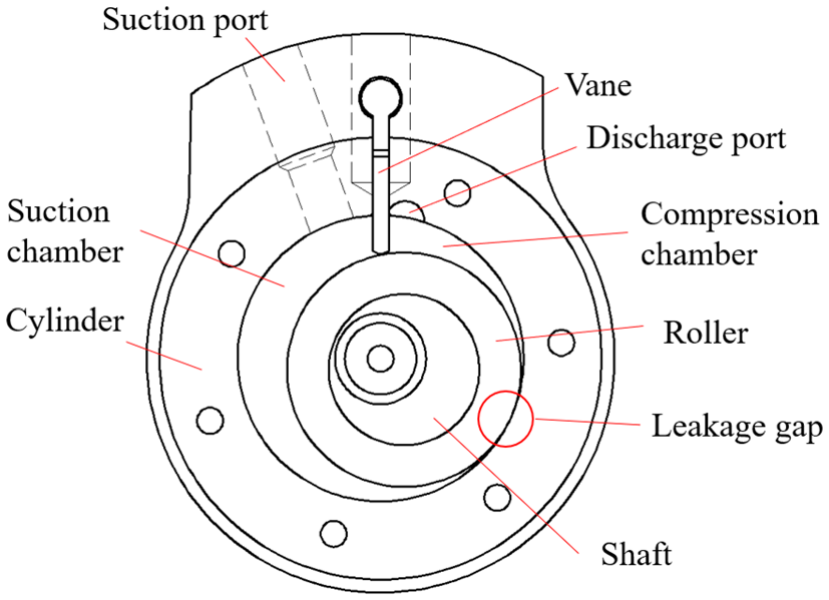
Geometry model of the working chambers.

The geometry model of the working chambers of the rotary compressor is shown in the Fig. 1. The chambers are mainly composed of a roller, a vane, a cylinder and two bearings above and below the cylinder. The roller is mounted eccentrically in the cylinder, and both them have the same rotation center. With the rotation of the roller, the low-pressure refrigerant gas flows into the suction chamber through the suction port. At the same time, the refrigerant in the compression chamber is compressed to the state of high pressure. When the pressure in the compression chamber increases to the discharge pressure, refrigerant flows into the shell through the discharge port. The high-pressure gas leaks from the compression chamber to the suction chamber through the leakage gap.

**Figure 2:**
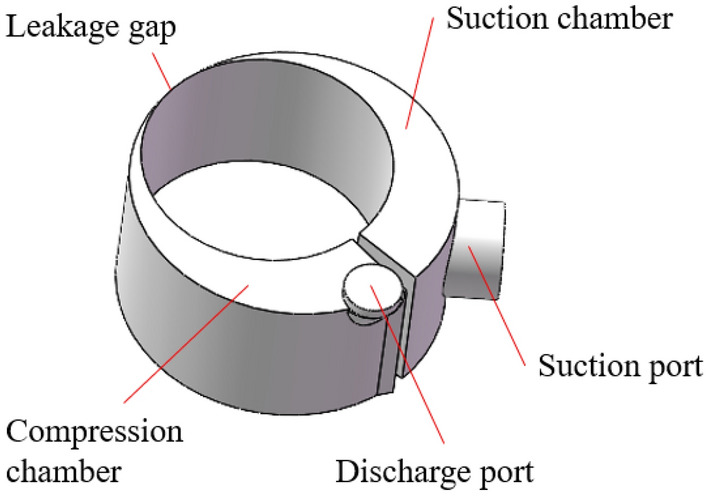
3D model of the fluid domain.

Figure 2 shows the 3D model of the fluid domain in the cylinder, which includes the suction port, the suction chamber, the compression chamber and the discharge port.

### Meshing and dynamic mesh model

**Figure 3:**
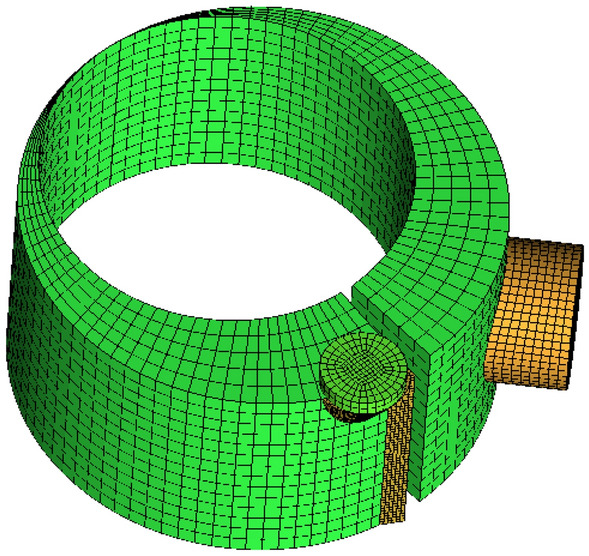
Computational grid.

The ANSYS ICEM is used to mesh the computational domain and most are structured grids to improve the mesh quality and reduces the mesh size. The computational grid is shown in Fig. 3.

In order to simulate the eccentric rotary motion of the roller in the cylinder, the User Defined Functions DEFINE_GRID_MOTION is used to control the node coordinates. The motion trajectory of the roller is simplified to a cycloid motion. The mathematical equations of a node are:

1 $$(x_{r} - e\cos (\theta ))^{2} - (y_{r} - e\sin (\theta ))^{2} = r_{r}^{2}$$

2 $$x_{c}^{2} - y_{c}^{2} = r_{c}^{2}$$

3 $$\frac{y}{x} = \frac{{y_{r} }}{{x_{r} }} = \frac{{y_{c} }}{{x_{c} }} = const$$

4 $$\frac{{\sqrt {x^{2} + y^{2} } - \sqrt {x_{c}^{2} + y_{c}^{2} } }}{{\sqrt {x_{r}^{2} + y_{r}^{2} } - \sqrt {x_{c}^{2} + y_{c}^{2} } }} = const$$

where (*x*, *y*) is the node coordinate. (*x*_*r*_, *y*_*r*_) is the innermost node coordinate along radial direction on the roller. (*x*_*c*_, *y*_*c*_) is the outermost node coordinate along radial direction on the cylinder. Coordinate origin of all nodes is the center of the cylinder. *e* is the eccentricity of the roller. *θ* is the eccentric angle, which can be calculated by angular velocity and current time. *r*_*c*_ is the cylinder radius. *r*_*r*_ is the piston radius.

**Figure 4:**
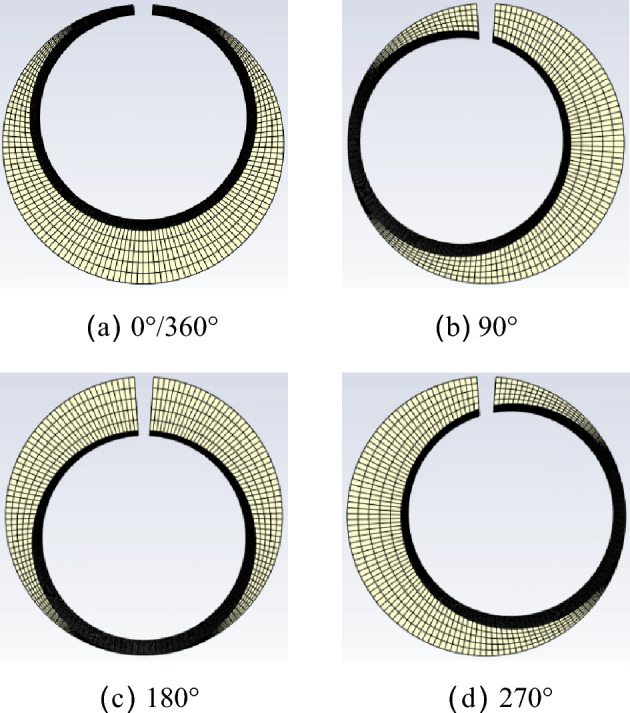
Mesh deformation at different angles.

The current node coordinates are obtained based on the previous node coordinates and the time step. To ensure the grid quality at the leakage gap, the grid elements only stretch and compress. The deformation of the mesh at different angles is shown in Fig. 4.

### Boundary conditions

The inlet boundary condition at the suction port is set as pressure inlet, which can be given by evaporating pressure. The volume fraction of the second phase is set according to calculation needs.

Due to lacking the discharge valve in the model and no reverse flow setting in the two-phase flow, the discharge port is set as wall condition at the beginning, and pressure outlet when the pressure at the discharge port reaches condensing pressure. The operation conditions are shown in Table 1. The main geometry parameters of the rotary compressor is shown in Table 2.

**Table 1:** Operating conditions.

Ref	*T*_*evap*_/°C	*P*_*evap*_/MPa	*T*_*cond*_/°C	*P*_*cond*_/MPa	Freq/Hz
R290	7.2	0.58761	46	1.5690	60
R32	7.2	1.0177	46	2.8616	60

**Table 2:** Main geometry parameters of the rotary compressor.

Parameter	Cylinder diameter/mm	Stroke volume/cm^3^	Cylinder height/mm	Eccentricity/mm
Value	50	17.9	24	5.3

### Fundamentals of CFD method

The Euler model of two-phase flow is used in this paper due to the predominant simulation performance for the interaction and separation in two-phase flow. The Euler model should determine the diameter of the second phase, and there is no direct measurement of the diameter of the droplet in rotary compressor in the existing papers. Therefore, referring to the relationship between the droplet diameter, frequency and inlet volume fraction in the multiphase rotary pump proposed in the paper^16^. In order to reflect the more serious liquid compression process, the droplet diameter is determined to be 100 μm.

The conservation equations of mass and momentum of the fluid “q”in this model are:

5 $$\frac{{\partial (\alpha_{q} \rho_{q} )}}{\partial t} + \nabla \cdot (\alpha_{q} \rho_{q} \vec{u}_{q} ) = \sum\limits_{q = 1}^{n} {\dot{m}_{pq} }$$

6 $$\frac{{\partial (\alpha_{q} \rho_{q} \vec{u}_{q} )}}{\partial t} + \nabla \cdot (\alpha_{q} \rho_{q} \vec{u}_{q} \vec{u}_{q} ) = - \alpha_{q} \nabla P + \nabla \tau + \alpha_{q} \rho_{q} g + R_{qp} + \dot{m}_{pq} \vec{u}_{pq}$$

where $\alpha_{q}$ and $\rho_{q}$ are the volume fraction and the density. $\vec{u}_{q}$ is the velocity. $\dot{m}_{pq}$ and $R_{qp}$ are the mass transfer rate and interphase drag from *p*_*th*_ phase to *q*_*th*_ phase. P is the pressure,$\nabla \tau$ is the pressure strain tensor. g is the gravitational acceleration.

Considering the computational resources for calculating the two-phase flow in the cylinder and the calculation accuracy, the SST k-*w* turbulence model is used in this paper. The Reynolds stresses is calculated by average velocity gradients and eddy viscosity in the model:

7 $${ - }\rho \mathop {u_{i} u_{j} }\limits^{\_\_\_\_} = \mu_{t} \left( {\frac{{\partial U_{i} }}{{x_{j} }} + \frac{{\partial U_{j} }}{{x_{i} }}} \right) - \frac{2}{3}\delta_{ij} \left( {\rho \kappa + \mu_{t} \frac{{\partial U_{k} }}{{x_{k} }}} \right)$$

where $\mu_{t}$ is the eddy viscosity, $\kappa$ is the turbulence kinetic energy. $\mu_{t}$ is given this equation:

8 $$\mu_{t} { = }\rho \frac{\kappa }{w}$$

where *w* is the turbulence frequency.

The phase change model is used in the calculation. This model defines the phase change rate based on the heat transfer and the overall heat balance at the phase interface. And the phase change coefficient doesn’t need to adjust due to the stability of the mass transfer source to the numerical solution.

The phase interface is in thermal equilibrium:

9 $$Q_{g} + Q_{l} = 0$$

where ${Q}_{l}$ is the heat transfer from phase interface to liquid and ${Q}_{g}$ is the heat transfer from phase interface to gas, which are given by:

10 $$Q_{l} = h_{l} A(T_{sat} - T_{l} ) - \dot{m}_{gl} \cdot H_{l}$$

11 $$Q_{g} = h_{g} A(T_{sat} - T_{g} ) + \dot{m}_{gl} \cdot H_{g}$$

where subscript *g*, *l*, *sat* represent gas, liquid and saturation respectively. *h* is the heat transfer coefficient. *H* is the enthalpy.* A* is the heat transfer area between gas and liquid. *T* is the temperature and $\dot{m}_{gl}$ is the phase transition rate.

The phase change rate can be calculated by the above three equations:

12 $$\dot{m}_{gl} = \frac{{h_{g} A(T_{sat} - T_{g} ) + h_{l} A(T_{sat} - T_{l} )}}{{H_{g} - H_{l} }}$$

The heat transfer coefficient between the gas and liquid is selected as two-resistance, where the heat transfer coefficient of the gas is ranz-marshall, and the liquid phase is zero-resistance.

The coupled pressure–velocity coupling method is chosen due to the great physical properties changing in the compression progress and the strong calculation nonlinearity. The flow courant number is 1. The explicit relaxation factor is 0.5, and the rest are default settings. The time step during calculation is adaptively adjusted, and the adjustment method is Multiphase-Specific, and the Global Courant Number is 2.

## Results and discussion

### Pressure and gas fraction field distributions

**Figure 5:**
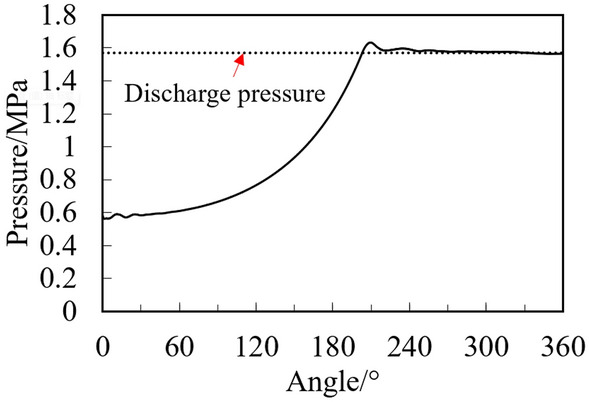
Pressure change in the compression chamber with angle.

Under the rated cooling condition as shown in Table 1, the two-phase compression of R290 in the cylinder with the initial gas fraction of 0.9 is simulated. The initial liquid is uniformly distributed in the cylinder chambers. Figure 5 shows the pressure change in the compression chamber in one cycle at the point near discharge port. The pressure change in the compression chamber is basically the same as that of single-phase gas compression, because of the small initial liquid volume fraction (about 0.0025), The pressure fluctuation only happens at the angle of beginning discharge with the maximum value of 0.061 MPa higher than the discharge pressure.

**Figure 6:**
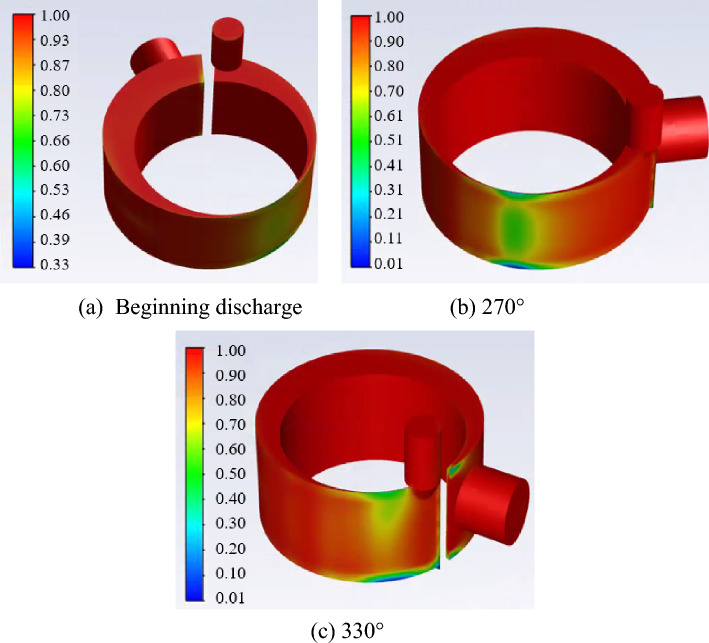
Gas fraction distribution in the cylinder.

Figure 6 shows the gas fraction distribution in the cylinder at the crank angle of beginning discharge, 300° and 330° respectively. It can be found from the figures that most of the liquid accumulates at the bottom of the chamber or the gap between the piston and the cylinder, due to the higher gravity and inertia force of the liquid than that of the gas. In the late discharge process in Fig. 6c, most liquid gathers at the bottom of the chamber near the discharge port. It can also be noted from the figure that the liquid concentration in the compression chamber increases with the increase of the angle.

**Figure 7:**
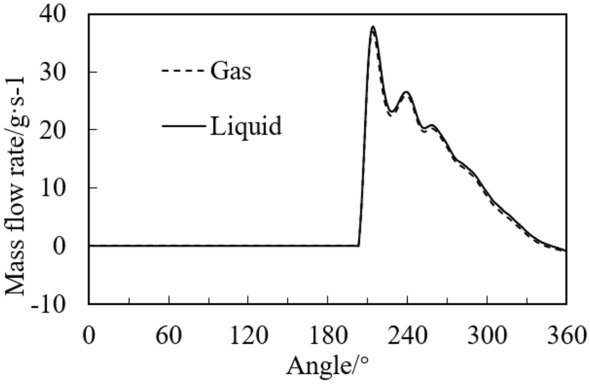
Mass flow rate of gas and liquid at the discharge port with angle.

Figure 7 shows the mass flow rate of gas and liquid flowing out of the discharge port with angle. It can be seen from the figure that both the gas and liquid begin to discharge at the angle about 200°, and the mass flow rates of them are basically equal. Since the density of the liquid is much greater than that of the gas, the volume flow rate of the liquid is ten times smaller than that of the gas. The mass flow rate of gas and liquid increases rapidly after the discharge valve opens, and the maximum value is about 37 g/s. The mass flow rates go down with the increase of the angle, because of the decrease of volume change rate of the cylinder.

### Effect of initial liquid volume fraction on two-phase compression in the cylinder

Based on the rated cooling conditions shown in Table 1, the gas–liquid two-phase compression characteristics of R290 in the cylinder is simulated at a high frequency of 120 Hz with the initial liquid volume fraction of 0.1, 0.2 and 0.3, respectively.

**Figure 8:**
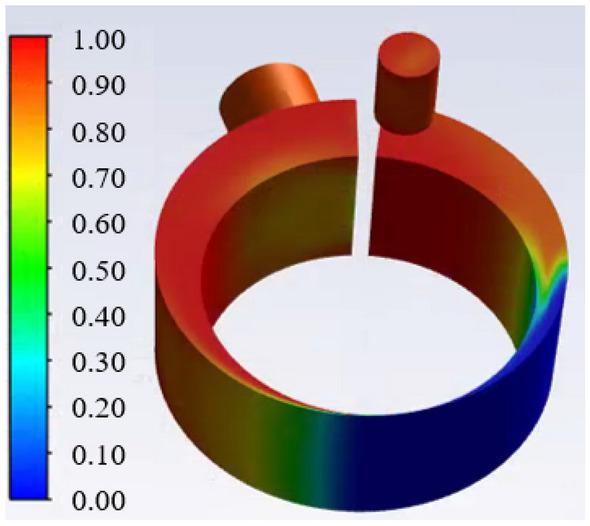
Gas fraction distribution in the cylinder.

**Figure 9:**
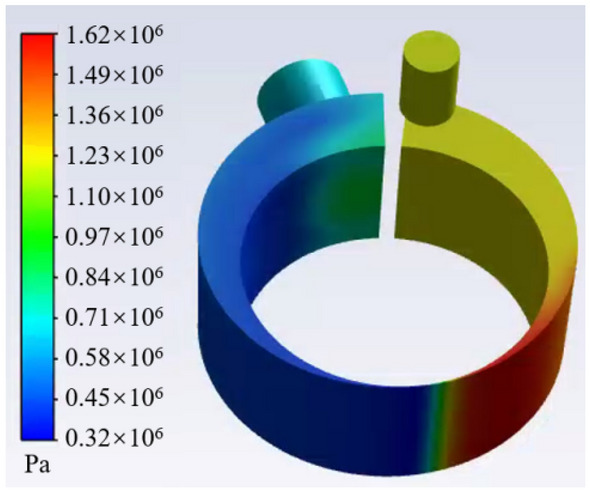
Pressure distribution in the cylinder.

Figures 8 and 9 show the gas fraction distribution and the pressure distribution in the cylinder at the angle of beginning discharge when the liquid volume fraction is 0.1. It can be found from the Fig. 8 that the most liquid refrigerant in the compression chamber is closer to the leakage gap than the gas due to the high density of the liquid. The uneven distribution of gas and liquid refrigerant results in a significant pressure gradient. Because much liquid accumulates near the leakage gap, its pressure is about 3.2% higher than the pressure near the discharge port.

**Figure 10:**
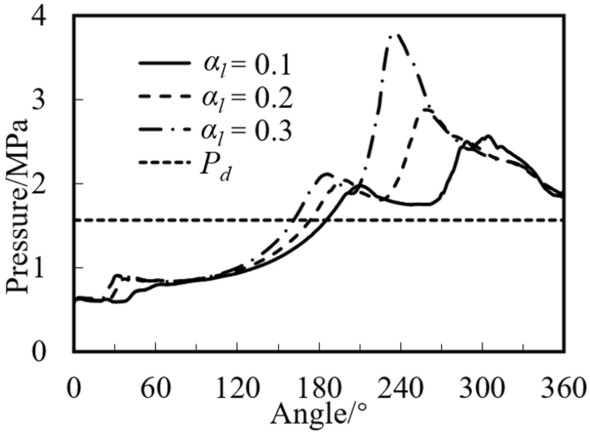
Variations of maximum pressure in the cylinder at different crank angles.

Under different initial liquid volume fractions, variations of the maximum pressure in the compression chamber near the leakage gap at different crank angles are shown in Fig. 10.

It can be noted that the maximum pressure value increases with the increase of the liquid volume fraction. When the liquid volume fraction is 0.3, 0.2, and 0.1, the pressures are 2.566, 2.884, and 3.798 MPa, which are 64%, 84%, and 142% higher than the discharge pressure, respectively. It can also be found that with the increase of the liquid volume fraction, the angle of the peak pressure decreases. The angle of the peak pressure decreases with the decrease of the liquid volume fraction. When the liquid volume fraction is 0.3, 0.2, and 0.1, the angle of the peak pressure is 304°, 258°, and 236°, respectively.

**Figure 11:**
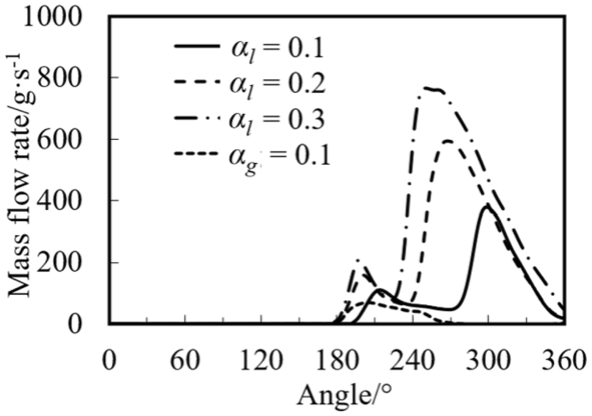
Variations of mass flow rate under different initial volume fractions.

Figure 11 shows the variations of mass flow rate at the discharge port with crank angle under different liquid volume fractions. Since the mass flow rates of the gas for three cases are almost equal, the gas mass flow rate with the fraction of 0.1 is displayed. It can be found from the figure that the mass flow rate of the liquid is much greater than that of the gas, and the gas flow rate reaches the maximum of 71 g/s at 193°. When the liquid volume fraction is 0.3, 0.2, and 0.1, the maximum flow rate of the liquid is 378, 594, and 765 g/s, respectively. The angle of the maximum flow rate is equal to that of the maximum pressure (see Fig. 10). Both variations of the pressure and the liquid mass flow rate have two peaks, which is consistent with the experimental results in the literature^11^. In general, even with high initial liquid volume fraction, the maximum pressure in the cylinder during liquid compression process for the rotary is small and does not exceed 3 times the discharge pressure.

### Effect of refrigerant type on two-phase compression in the cylinder

Based on the rated cooling conditions shown in Table 1, the gas–liquid two-phase compression characteristics of R290 and R32 in the cylinder with the initial gas fraction of 0.9 are simulated. Due to the high initial gas fraction, no secondary pressure rise in the compression chamber is found during the simulation.

**Figure 12:**
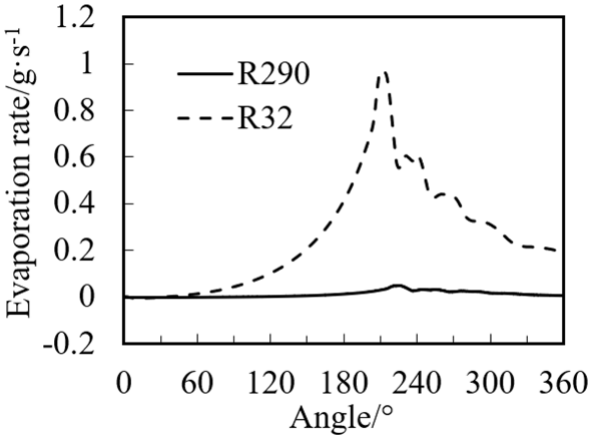
Evaporation rate of refrigerant at different angles in the compression chamber.

**Figure 13:**
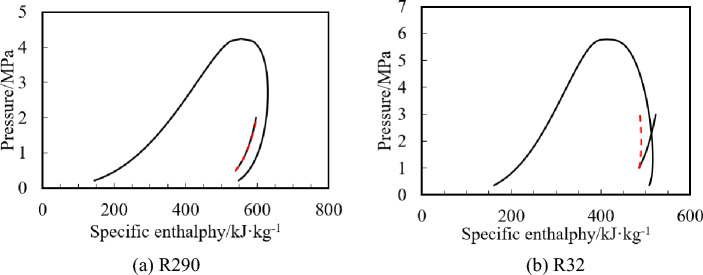
Isentropic and isothermal lines of R290 and R32.

Figure 12 shows the average evaporation rate of refrigerant at different angles in the compression chamber. Figure 13 shows the isentropic and isothermal lines of R290 and R32 under the same operating conditions. The dotted line in the figure is the isotonicity line, and the solid line is the isentropic line.

It can be seen from Fig. 12 that with the increase of the angle, the evaporation rate increases first and then decreases, and reaches the maximum value at the end of compression process of about 200°. This is because the pressure and the saturation temperature of the refrigerant decline with the decrease of compression chamber volume due to the increase of the angle. The actual refrigerant temperature is much higher than the saturation temperature, so the evaporation rate enhances.

It can also be seen from Fig. 12 that the evaporation rate of R32 is much higher than that of R290. At the end of compression (about 200°), the maximum evaporation rate of R290 is 0.047 g/s, while that of R32 is 0.969 g/s, which is about 20 times as much as that of the R290. This is because, as shown in Fig. 13, the slopes of isentropic line and the isothermal line of R290 with the initial gas fraction of 0.9 are almost equal. However, the slope of isentropic line of R32 is much less than that of the isothermal line. This results in the greater enthalpy increase and the faster evaporation rate of the R32 than that of R290 under the same adiabatic two-phase compression condition. After opening the discharge valve, the refrigerant pressure in the cylinder no longer increases, so the evaporation rate slows down.

**Figure 14:**
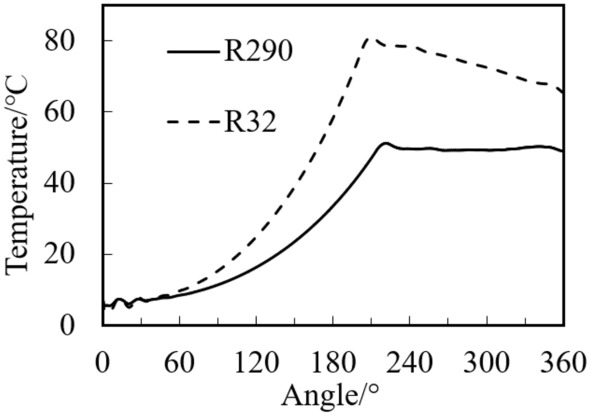
Variations of temperature with crank angle.

Figure 14 shows the variations of R290 and R32 temperature with crank angle. It can be noted that temperature of R32 is much greater than that of R290 during both compression and discharge processes. The temperature of R32 during the discharge process decreases with the increase of angle because of much liquid evaporation (see Fig. 12), while the temperature of R290 during this process changes little due to little liquid evaporation.

### Effect of refrigerant type on two-phase compression in the cylinder

**Figure 15:**
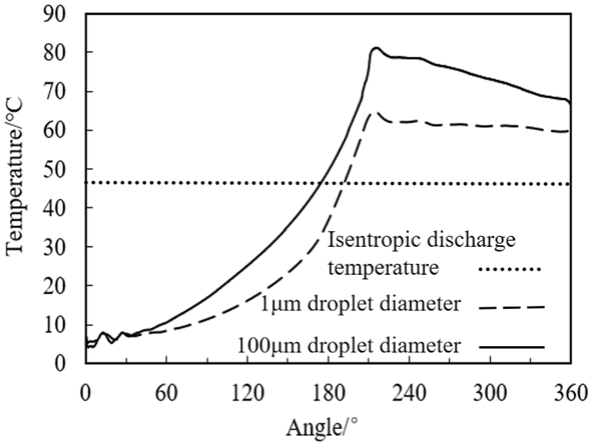
Temperature change with different droplet diameters.

Figure 15 shows the temperature change with different droplet diameters. By injecting the liquid refrigerant into compressor to reduce the droplet diameter as much as possible, the temperature of the compression chamber can be effectively reduced and the pressure of the compression chamber will not be higher. As shown in the figure, when the droplet diameter is 1 μm, the temperature in the compression chamber is 16℃ lower than that of the droplet diameter of 100 μm, because the small droplet diameter increases the gas–liquid phase heat transfer area. Compared with the isentropic discharge temperature, the discharge temperature is about 18℃ higher than the droplet diameter of 1 μm. This is because, even if the droplet diameter is only 1 micron, some droplets are not evaporated.

**Figure 16:**
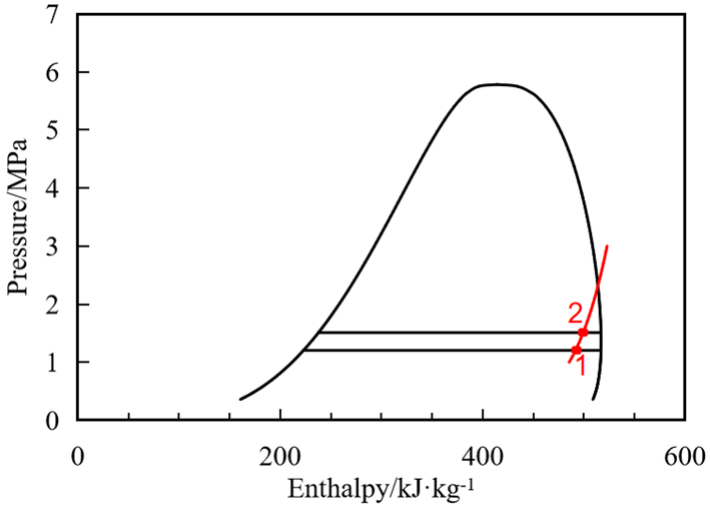
Isentropic compression process of the R32.

Figure 16 shows the isentropic compression process of the R32 in the Pressure-enthalpy diagram, which assumes no leakage between suction chamber and compression chamber.

Assuming the refrigerant is compressed from state 1 to state 2, and the mass can be obtained:

13 $$m_{g1} + m_{l1} = m_{g2} + m_{l2} = m_{mix}$$

where subscript *g*, *l* represent gas, liquid. m is the mass.

The mass can be obtained from the respective volume and density:

14 $$m_{g,l} = \rho_{g,l} V_{g,l}$$

where* ρ* is the density, V is the volume.

The sum volume of gas and liquid can be obtained from the angle of the roller:

15 $$V_{g} + V_{l} = f(\theta )$$

where *θ* is the angle of the roller.

The isentropic compression process from state 1 to 2 can be expressed as bellow:

16 $$\frac{{m_{g1} }}{{m_{{{\text{mix}}}} }}s_{g1} + \left( {1 - \frac{{m_{g1} }}{{m_{{{\text{mix}}}} }}} \right)s_{l1} = \frac{{m_{g2} }}{{m_{{{\text{mix}}}} }}s_{g2} + \left( {1 - \frac{{m_{g2} }}{{m_{{{\text{mix}}}} }}} \right)s_{l2}$$

where subscript *mix* represents mixture of gas and liquid. s represents entropy.

Since the compression is in the two-phase region at this time, the physical properties of the gas–liquid phase can be determined by temperature:

17 $$(\rho_{g} ,\rho_{l} ,s_{g} ,s_{l} ) = f(T)$$

Combining the Eqs. (13) and (16), the gas mass at state 2 is :

18 $$m_{g2} = \frac{{V_{2} - \frac{{m_{mix} }}{{\rho_{l2} }}}}{{\frac{1}{{\rho_{g2} }} - \frac{1}{{\rho_{l2} }}}}$$

Combining the Eqs. (17) and (18), the current temperature T can be obtained. According to the Eq. (18) the current gas and liquid mass can be obtained, and then the evaporation rate can be obtained.

**Figure 17:**
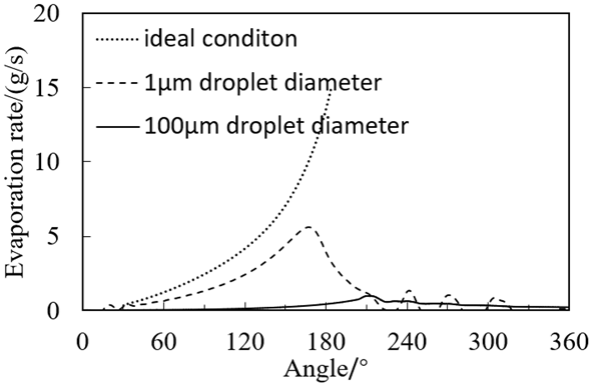
Droplets evaporation rate with different diameters during compression processes.

The above deduction is based on the ideal evaporation state. The comparison between ideal result and CFD calculation result is shown in Fig. 17. In the compression process, the evaporation rate accelerates with the increase of the angle. When the droplet diameter is 100 μm and the droplet diameter is 1 μm, the maximum evaporation rate increases from 0.97 g/s to 5.6 g/s, accelerating by about 6 times. While the ideal evaporation state evaporation rate can reach the maximum of 15 g/s, when all the liquid is evaporated, which is 2.7 times higher than that when the droplet diameter is 1 μm. Therefore, even if the droplet diameter is small enough, due to the local aggregation of the liquid during actual evaporation, the temperature gradient exists in the compression chamber, and the liquid can’t evaporate as the ideal state. That is, the two-phase compression in the cylinder does not meet the isentropic compression state even if the wall insulation.

### Effects of compressor type on two-phase compression in the cylinder

**Figure 18:**
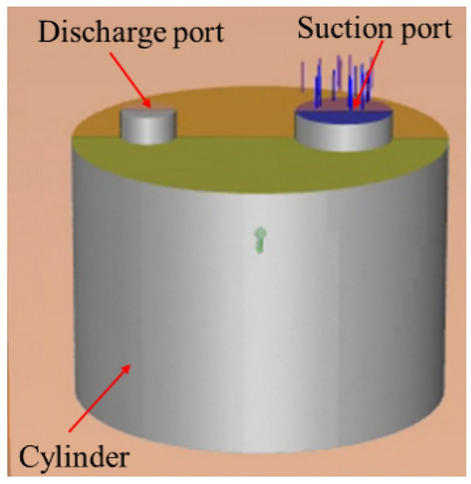
The forward sectional view of the reciprocating compressor.

The two-phase compression in a reciprocating compressor and a rotary compressor are simulated under the rated cooling condition show in Table 1 with the initial liquid volume fraction of 0.1. Table 3 shows the dimensions of the reciprocating compressor, which has the same stroke volume as the rotary compressor. The forward sectional view of the compressor along the cylinder, suction and discharge ports axis is shown in Fig. 18.

**Table 3:** Dimensions of the reciprocating compressor.

D_cylinder_/mm	Stroke volume/mm	δ_dead_point_/mm	L_crank_/mm	D_suc_/mm	D_dis_/mm
27	18	1	34	7	4

**Figure 19:**
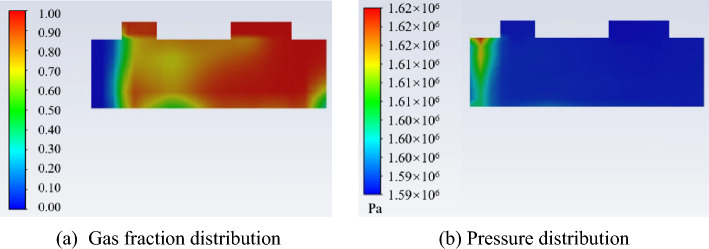
Pressure distribution and gas fraction distribution at the crank angle of beginning discharge.

Figure 19 shows the pressure distribution and gas fraction distribution in the cylinder at the crank angle of beginning discharge. It can be found from Fig. 19a that most of the liquid during the compression process is accumulated under the discharge port, which leads to the higher pressure under the discharge port than that of the suction port as shown in Fig. 19b. The reason for the liquid distribution is that, the liquid moves more slowly than the gas with the movement of the piston due to the high liquid density.

**Figure 20:**
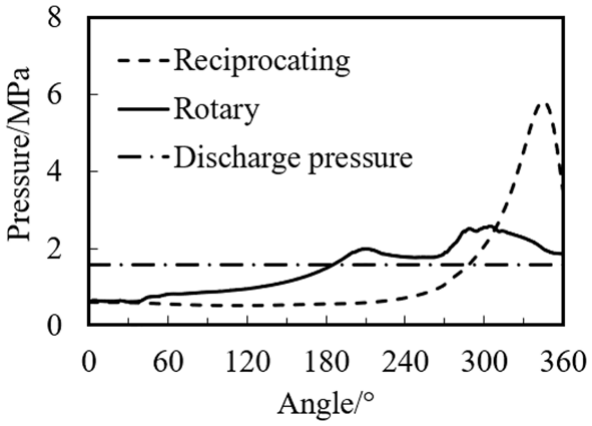
variations of the pressure with crank angle.

Figure 20 shows the variations of the pressure in the cylinder of the rotary compressor and the reciprocating compressor with crank angle under the same operating conditions.

It can be found from the Fig. 20 that after the compression starts, the pressure of the reciprocating compressor is greater than that of the rotary compressor. The maximum pressure of the rotary compressor is 2.56 MPa at the crank angle of 304°. The maximum pressure of the reciprocating compressor is 5.799 MPa at the crank angle of 345°, which is 2.26 times higher than that of the rotary compressor. There are mainly two reasons for this phenomenon. First, the compression and discharge process of the reciprocating compressor is 180° (from the crank angle of 180° to 360°), which is half of the rotary compressor. Much more refrigerant has to be discharged within smaller angles for the reciprocating compressor. Second, the piston motion direction and discharge direction between the two compressors are different. The piston motion direction and the discharge direction of the reciprocating compressor are both along the axis of the cylinder. The piston motion direction of the rotary compressor is radial of the cylinder, and the discharge direction is axial of the cylinder, which results in a smaller discharge resistance of the rotary compressor under the same conditions.

**Figure 21:**
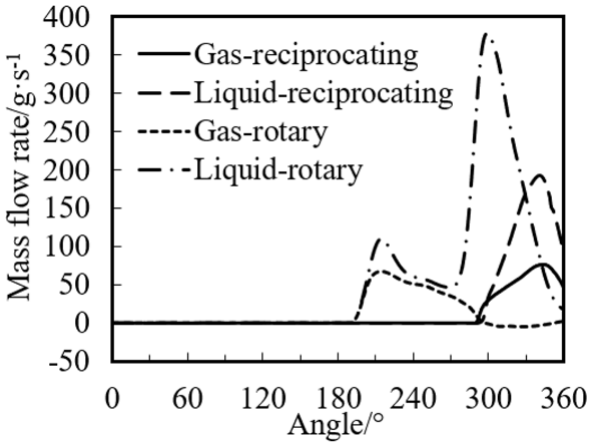
Variations of the discharge mass flow rate with crank angle.

Figure 21 shows the variations of the discharge mass flow rate of the rotary compressor and the reciprocating compressor with crank angle. It can be found from the figure that the maximum gas discharge rates for the two compressors are close, while the liquid discharge rate of the rotary compressor is much higher than that of the reciprocating compressor. The maximum liquid discharge rate of the reciprocating compressor is 193 g/s at 3441°, and that of the rotary compressor is 378 g/s at 299°, which is 1.96 times of the reciprocating compressor.

It can also be found from Fig. 21 that much liquid is discharged from the cylinder of the rotary compressor during the discharge process, while less liquid is discharged for the reciprocating compressor discharges. So there is more liquid left in the cylinder of the reciprocating compressor under the same two-phase compression conditions, which results in the severe liquid slugging.

## Conclusion

In this paper, the numerical simulation of the liquid–vapor two-phase refrigerant compression in the cylinder of rotary compressors is carried out using the CFD method based on commercial software ANSYS Fluent. Dynamic mesh and phase change model are considered in the simulation. Effects of initial liquid volume fraction, refrigerant types, and compressor types on the two-phase characteristics are conducted. The following conclusions may be drawn:

1. Under the condition of a small amount of liquid compression, the discharge pressure of the rotary compressor is basically the same as that of the single-phase compression. During the two-phase compression process, most liquid is located near the leakage gap and at the bottom of the compression chamber.
2. Under the low initial liquid volume fraction conditions, two peak pressures appear during the discharge processes. The peak pressure decreases with the increase of the liquid volume fraction. The peak pressures are 64%, 84%, and 142% higher than the discharge pressure with the liquid volume fraction of 0.3, 0.2, and 0.1. This high peak pressure will not cause damage to the compressor.
3. Under the same two-phase compression conditions, the evaporation rate of R32 in the cylinder is much higher than that of R290, and the temperature of R32 decreases faster during the discharge process than that of the R290.
4. Reciprocating piston compressors are more prone to liquid slugging than rotary compressors. Under the low initial liquid volume fraction, the maximum pressure of the reciprocating compressor is 2.26 times higher than that of the rotary compressor.
